# Functional Identification of Api5 as a Suppressor of E2F-Dependent Apoptosis In Vivo

**DOI:** 10.1371/journal.pgen.0020196

**Published:** 2006-11-17

**Authors:** Erick J Morris, William A Michaud, Jun-Yuan Ji, Nam-Sung Moon, James W Rocco, Nicholas J Dyson

**Affiliations:** 1 Massachusetts General Hospital Cancer Center, Laboratory of Molecular Oncology, Charlestown, Massachusetts, United States of America; 2 Harvard Medical School, Boston, Massachusetts, United States of America; 3 Division of Surgical Oncology, Massachusetts General Hospital, Boston, Massachusetts, United States of America; 4 Massachusetts Eye and Ear Infirmary, Boston, Massachusetts, United States of America; Fred Hutchinson Cancer Research Center, United States of America

## Abstract

Retinoblastoma protein and E2-promoter binding factor (E2F) family members are important regulators of G1-S phase progression. Deregulated E2F also sensitizes cells to apoptosis, but this aspect of E2F function is poorly understood. Studies of E2F-induced apoptosis have mostly been carried out in tissue culture cells, and the analysis of the factors that are important for this process has been restricted to the testing of a few candidate genes. Using *Drosophila* as a model system, we have generated tools that allow genetic modifiers of E2F-dependent apoptosis to be identified in vivo and developed assays that allow effects on E2F-induced apoptosis to be studied in cultured cells. Genetic interactions show that dE2F1-dependent apoptosis in vivo involves dArk/Apaf1 apoptosome-dependent activation of both initiator and effector caspases and is sensitive to levels of *Drosophila* inhibitor of apoptosis-1 (dIAP1)*.* Using these approaches, we report the surprising finding that apoptosis inhibitor-5/antiapoptosis clone-11 (Api5/Aac11) is a critical determinant of dE2F1-induced apoptosis in vivo and in vitro. This functional interaction occurs in multiple tissues, is specific to E2F-induced apoptosis, and is conserved from flies to humans. Interestingly, Api5/Aac11 acts downstream of E2F and suppresses E2F-dependent apoptosis without generally blocking E2F-dependent transcription. Api5/Aac11 expression is often upregulated in tumor cells, particularly in metastatic cells. We find that depletion of Api5 is tumor cell lethal. The strong genetic interaction between E2F and Api5/Aac11 suggests that elevated levels of Api5 may be selected during tumorigenesis to allow cells with deregulated E2F activity to survive under suboptimal conditions. Therefore, inhibition of Api5 function might offer a possible mechanism for antitumor exploitation.

## Synopsis

The retinoblastoma protein (pRB) was the first human tumor suppressor to be described, and it works by limiting the activity of the E2F transcription factor. The pRB pathway is inactivated in most forms of cancer, and, accordingly, most tumor cells have deregulated E2F. Uncontrolled E2F drives cell proliferation, but it also sensitizes cells to die (apoptosis). E2F-induced apoptosis is not well understood, but it affects the development of cancer and, potentially, could be exploited for cancer treatment. To date, however, there have been very few studies of E2F-induced apoptosis in animal models. The authors describe a series of genetic tools that allow systematic studies of E2F-induced apoptosis in *Drosophila*. As validation, this approach identified some known regulators of E2F-dependent apoptosis and also identified *Api5,* a little-studied gene that had not previously been linked to E2F, as a potent suppressor of E2F-induced cell death. The effects of *Api5* on E2F occur in several different tissues and are conserved from flies to humans. This last point is significant since *Api5* is upregulated in cancer cells. The discovery of the E2F–*Api5* interaction demonstrates that important modulators of E2F-induced apoptosis are waiting to be discovered and that they can be found using *Drosophila*.

## Introduction

A proper balance between cell proliferation and apoptosis is crucial for organism development and function. Perturbations in this balance underlie a variety of pathological conditions, including cancer (for review, see [1]). E2-promoter binding factor (E2F) family proteins are important regulators of cell cycle progression and a major target for regulation by the retinoblastoma protein (pRB) tumor suppressor protein family (for review, see [2,3]). The pRB pathway is functionally inactivated in most tumor cells, and the resulting change in E2F activity is thought to allow unchecked cell proliferation.

In addition to their ability to drive cell proliferation, E2F proteins sensitize cells to apoptosis (for review, see [4,5]). E2F-induced apoptosis limits the consequences of E2F deregulation to such an extent that tumorigenesis selects not only for lesions in the pRB pathway but also for mutations that suppress the apoptotic potential of E2F (for review, see [6,7]). Studies in mice show that apoptosis significantly limits tumorigenic growth following pRB inactivation, and the cell types that are most prone to tumorigenesis following the inactivation of pRB-family members are those that are intrinsically resistant to apoptosis [8–10].

While the effects of E2F on the control of cell cycle progression are well known, the connections between E2F and the apoptotic machinery are less well defined. E2F-induced apoptosis is a property associated with some E2F family members (notably mammalian E2F1 and *Drosophila* dE2F1) but not with others [11,12]. After acute DNA damage in mammalian cells, E2F1 is selectively modified and activates transcription from a subset of E2F-regulated promoters, resulting in activation of a large number of apoptotic regulators (for review, see [13]). However, the relative importance of these targets appears to be context dependent. In various studies, E2F1-induced apoptosis has been reported to be *p53* dependent, *p53* independent, *Apaf1* dependent, *Apaf1* independent, and *p73* dependent [14–22]. Other studies have found E2F1-induced apoptosis to be inhibited by the expression of either pRB [23] or TopBP1 [24], by the addition of serum [25], or by the activation of Akt signaling [26]. The large number of E2F-inducible genes, together with inconsistencies between studies carried out in different cell lines, raises the issue of whether there is one general mechanism of E2F-induced apoptosis or whether E2F induces apoptosis in different ways in different cell types. Studies in *Drosophila* show that the overall impact of E2F regulation on the DNA-damage response in vivo varies greatly between cell types [27].

To date, the study of E2F-induced apoptosis has been carried out primarily in tissue culture cells, and the analysis of the factors that are important for this process has been restricted to the testing of a few candidate genes. Because of this, it is highly likely that many of the genes that are most important for E2F-induced apoptosis in vivo have yet to be identified. To identify these genes, a genetic screening approach is required. Previous efforts have concentrated on E2F-stimulated proliferation [28] and, thus far, the genetic tools needed to study E2F-induced apoptosis have not been described.

To fill this void, we have exploited the Gal4/UAS misexpression system in *Drosophila* to generate transgenic lines with dosage-sensitive phenotypes that are caused by dE2F1-induced cell death. Here we describe these stocks and their utility to identify mutations that modify the extent of E2F-induced apoptosis. Moreover, we link this in vivo approach with in vitro studies in *Drosophila* cultured cells designed specifically to validate the genetic interactions on apoptosis per se. Using these reagents we show that apoptotic inhibitor-5/antiapoptosis clone-11 (Api5/Aac11), a highly conserved family of antiapoptotic proteins that have not previously been linked to E2F, function as strong and specific suppressors of E2F-dependent apoptosis. Api5/Aac11 is rate-limiting for E2F-induced phenotypes in *Drosophila* in multiple cell types and developmental contexts, and the genetic interaction between E2F and Api5/Aac11 is conserved between *Drosophila* and human cells. These results illustrate the value of genetic approaches for the study of E2F-dependent apoptosis, showing that despite the extensive molecular studies of the E2F transcriptional program, additional tiers of regulation exist that have a significant impact on E2F-induced processes in vivo.

## Results

### Generation of E2F-Dependent Phenotypes

**Figure 1:**
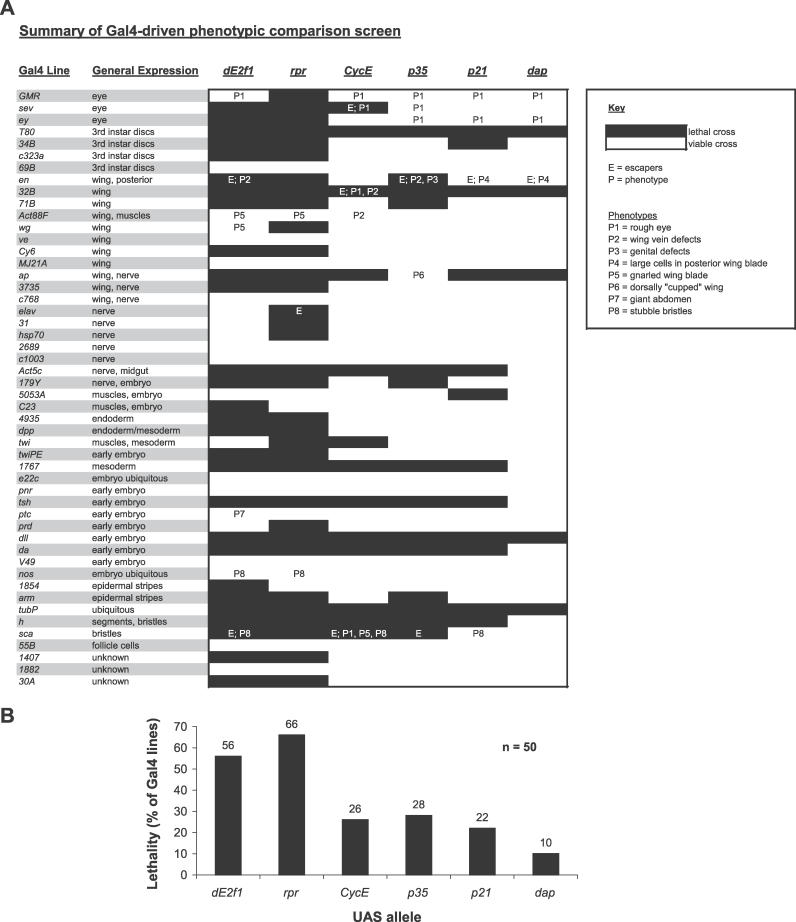
Summary of the Gal4-Driven Phenotypic Comparison Screen (A) Gal4 driver lines were crossed to various UAS alleles at 25 °C in order to identify novel dominant phenotypes. Phenotype modification was compared relative to control chromosome *w^1118^.* Crosses lethal to progeny are indicated in black, while viable crosses are indicated in white. Viable “escaper” flies from lethal crosses are indicated (E). Phenotypes generated in viable or escaper progeny are also indicated for each cross (P). (B) Expression of *rpr* and *dE2f1* resulted in significant lethality in the majority of Gal4 lines tested.

We sought visible phenotypes that were caused by E2F-induced apoptosis and were suitable for genetic screening. We used the Gal4-UAS system to express the *Drosophila E2f* gene *(dE2f1)* in a tissue-specific manner [29] and screened a collection of 50 Gal4 drivers that provided a broad assortment of developmentally regulated patterns. We compared the effects of expressing *dE2f1* with the effects of expressing known regulators of cell cycle progression such as *cyclin E (CycE), dacapo (dap),* and human *p21*, or apoptosis regulators such as *reaper (rpr)* or baculovirus caspase inhibitor, *p35* (Figure 1). Each of these transgenes caused lethality when combined with specific subsets of drivers, and in some cases gene expression resulted in visible phenotypes. Interestingly, in this general survey we noted that the consequences of expressing *dE2f1* closely paralleled the effects of expressing the *Drosophila* proapoptic gene, *rpr,* but showed far less similarity with the effects of expressing the cell cycle regulator *CycE*. Because we sought to study dE2F1-dependent apoptosis, we selected the combinations of transgenes in which *dE2f1* expression gave a visible phenotype that was phenocopied by the expression of *rpr,* but not by *CycE,* and we used these to generate stable stocks bearing dE2F1-dependent phenotypes.

**Figure 2:**
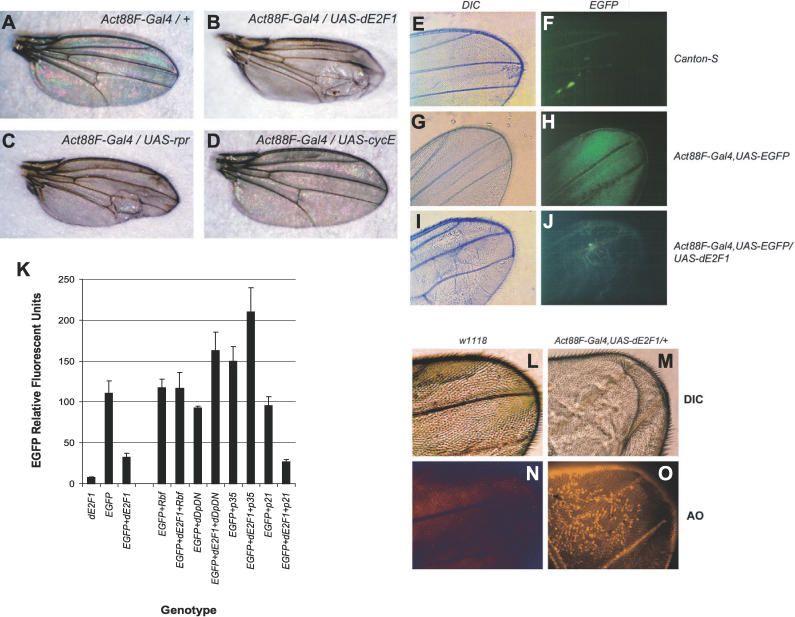
Generation and Characterization of Tissue-Specific dE2F1-Dependent Apoptotic Phenotypes in *Drosophila* The *Act88F-Gal4* driver was crossed to either a wild-type chromosome (*w^1118^; +*) (A), *UAS-dE2f1* (B), *UAS-rpr* (C), or *UAS-CycE* (D). Expression of *dE2f1* and *rpr*, but not *CycE*, was sufficient to induce a ventrally curved, blistered, and gnarled wing phenotype. In order to visualize expression patterns, *Act88F-Gal4* was crossed to either a wild-type chromosome (*Canton-S*) (E and F) or *UAS-EGFP* transgene (G and H). *Act88F* drives expression in the newly eclosed adult fly wing blade cells, and coexpression of *dE2f1* removes many EGFP-positive cells (I and J). EGFP expression was quantitatively determined in single transgenic flies by fluorescent spectrophotometry (K). Expression of *dE2f1* significantly reduced the levels of EGFP and was rescued by coexpression of *Rbf*, *dDpDN*, or *p35*, but not *p21*. Newly eclosed wild-type wings (L and N) or *dE2f1*-expressing wings (M and O) were stained with AO to visualize apoptotic cells. Cells expressing *dE2f1* had strong, punctuate AO staining in the distal wing region where *EGFP* is mainly expressed early after eclosion. DIC, differential interference contract.

We found several novel dE2F1-dependent phenotypes that gave stable stocks and appeared to be amenable to genetic screening (Figure 1). A dE2F1-dependent wing phenotype, generated by the *Drosophila Actin 88F (Act88F)* promoter, was particularly useful and was characterized in more detail (Figure 2). Transheterozygous crosses of *Act88F-Gal4* and *UAS-dE2f1* produced gnarled and ventrally curved wings that frequently contained blisters (Figure 2A and 2B). This phenotype was found to be 100% penetrant and was phenocopied by expression of other proapoptotic genes from the same driver, such as *rpr* (Figure 2C). The expression of the cell cycle genes *CycE* (Figure 2D) or *string/cdc25 (stg)* (unpublished data), in contrast, gave no significant wing curvature or gnarling.

The pattern of expression generated by the *Act88F* driver was visualized using a *UAS-enhanced green fluorescent protein (EGFP)* transgene. *Act88F* is expressed during the development of flight and thoracic muscles [30]. Consistent with this, the earliest *EGFP* expression was detected in indirect flight muscles of the pupae (unpublished data). In addition, we observed significant *EGFP* expression in cells of the newly eclosed wing blade (Figure 2E–2H). Coexpression of *dE2f1* resulted in significantly fewer *EGFP* expressing cells in the wing (Figure 2I–2J), a change that we quantified fluorometrically in single flies (Figure 2K).

To confirm that the reduction of EGFP-positive cells was due to apoptosis, wings of newly eclosed adults were stained with acridine orange (AO) to identify apoptotic cells. In wild-type discs, very few cells labeled with AO shortly after eclosion (Figure 2L and 2N). However, *dE2f1* expression caused a punctate pattern of AO staining within the blistered portion of the distal wing blade (Figure 2M and 2O). By 30 min after eclosion, both wild-type and *dE2f1*-expressing wings demonstrate significant levels of AO staining (unpublished data), consistent with previous reports of programmed cell death in the wing [31]. The punctate pattern of AO staining induced by dE2F1 was significantly different, and easily distinguishable, from the diffuse pattern of staining seen during the later wave of programmed cell death that occurs in the newly eclosed wing. These observations show that *dE2f1* expression induces premature cellular death, and we infer that these dE2F1-induced changes perturb the multicellular architecture of the wing epithelia, causing defects that become set in place during wing maturation.

**Figure 3:**
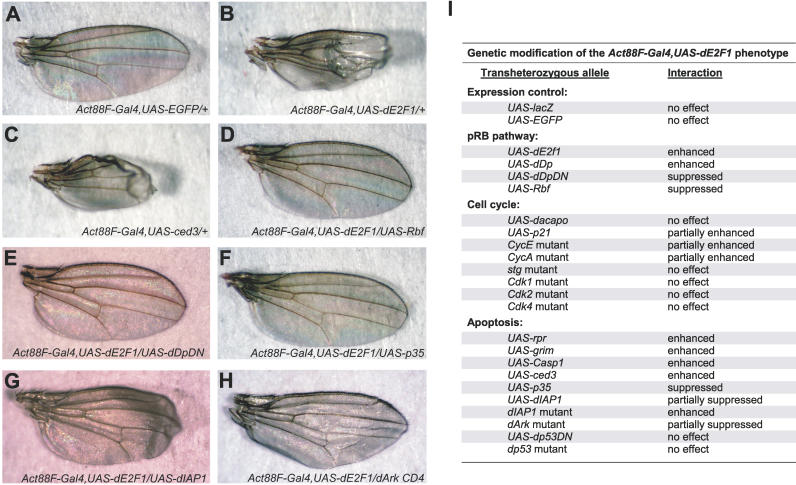
Genetic Characterization of a Recombinant *Act88F-Gal4,UAS-dE2f1* Transgenic Stock Various alleles were analyzed for modification of the *dE2f1-*dependent phenotype in trans. The wild-type wing phenotype is depicted in *Act88F-Gal4,UAS-EGFP/+ (w^1118^)* recombinant stock as control (A). The *Act88F-Gal4,UAS-dE2f1/+ (w^1118^)* recombinant stock phenotype (B) is strongly phenocopied by caspase expression (C). Coexpression of *Rbf* (D) or *dDpDN* (E) completely suppressed the *dE2f1* phenotype. The *dE2f1*-dependent phenotype was also suppressed by various apoptotic regulators including coexpression of the caspase inhibitor *baculovirus p35* (F) or *dIAP1* (G) or a heterozygous dominant allele of *dArk* (H), the *Drosophila APAF1* homolog. (I) Summary of the genetic interactions with *Act88F-Gal4,UAS-dE2f1.* The *Act88F-Gal4,UAS-dE2f1* recombinant chromosome was crossed at 25 °C to various transgenic and mutant alleles and phenotypes analyzed in transheterozygous progeny. Modification of the *dE2F1* phenotype was compared relative to control chromosome *w^1118^.* See Materials and Methods for mutant alleles used in this study.

As a further test of the processes involved in this dE2F1-induced phenotype, we crossed *Act88F-Gal4,UAS-dE2f1* recombinant stocks with various UAS and mutant alleles (Figure 3) and examined their genetic interactions. As expected, the wing phenotype was completely suppressed by the coexpression of the *Drosophila* pRB ortholog, *Rbf* (Figure 3D), or by the coexpression of a dominant-negative form of the dE2F1 heterodimerization partner, *dDp* (Figure 3E). These proteins also blocked dE2F1-induced apoptosis, as measured by the loss of *EGFP-*expressing cells (Figure 2K). Conversely, expression of additional *dE2f1* or the coexpression of functional *dDp* strongly enhanced the wing defects (Figure 3I). Taken together, with the suppression observed by dominant-negative *dDp*, these data strongly suggest that the wing phenotype is dependent on dE2F1-induced transcription. Control UAS transgenes, such as *UAS-EGFP* and *UAS-beta-galactosidase (lacZ),* had no effect on the *Act88F-Gal4,UAS-dE2f1* phenotype (Figure 3I).

We also tested for dominant interactions with various alleles of known cell cycle regulators (Figures 2K and 3I). Unlike *Rbf*, the expression of the human *p21* cyclin-dependent kinase inhibitor protein or the *Drosophila* p21 homolog *dap* failed to suppress the *Act88F-Gal4,UAS-dE2f1* phenotype. Loss-of-function mutations in *CycE, stg, Cdk1, Cdk2,* or *Cdk4* also failed to suppress the phenotype. Indeed, mutant alleles of *CycE* and the overexpression of *p21* caused a slight enhancement of the wing defects. Taken together, with the inability of *CycE* or *stg* to generate a similar phenotype, these results suggest that the *Act88F*-driven dE2F1-dependent wing phenotype is unlikely to be caused by cell cycle activation.

A clear pattern of strong genetic interactions emerged when alleles of apoptotic regulators were tested. The dE2F1*-*dependent phenotype was strongly suppressed by coexpression of the caspase inhibitors *baculovirus p35* or *Drosophila thread/inhibitor of apoptosis protein-1 (dIAP1)* (Figure 3F and 3G). Accordingly, caspase expression alone phenocopied the dE2F1 wing phenotype (Figure 3C). Caspase activity is triggered by activation of apoptosome complexes which regulate initiator caspase activation (for review, see [32]). Accordingly, heterozygous loss-of-function alleles in the *Drosophila Apaf-1-related killer (dArk)* were strong, dominant suppressors of the *Act88F-Gal4,UAS-dE2f1* phenotype (Figure 3H). In addition, loss-of-functional alleles of the endogenous caspase inhibitor *dIAP1* were strong enhancers of the phenotype (Figure 3I). These interactions indicate that apoptosome-caspase signaling is required for dE2F1-induced apoptosis and that this in vivo E2F-dependent phenotype can be dominantly modified by mutations in downstream apoptotic signaling pathways. Interestingly, *Act88F-Gal4,UAS-dE2f1* was unaltered by the expression of various dominant-negative alleles of *Drosophila p53 (dp53)* or by the introduction of mutant alleles of *dp53* (Figure 3I). Similar results were found with other dE2F1-dependent phenotypes (unpublished data). In mammalian cells, E2F-induced apoptosis can be either *p53* dependent or *p53* independent (for review, see [33]); in *Drosophila,* dE2F1-induced apoptosis appears to be primarily independent of *dp53*.

Taken together, these results show that expression of dE2F1 under the control of the *Act88F-Gal4* driver triggers cell death. *Act88F-Gal4,UAS-dE2f1* wings have a visible phenotype that can be readily modified by transgenes that affect E2F1 activity and by transgenes that induce or block cell death. Moreover, this phenotype can be dominantly enhanced or suppressed by heterozygous mutations in genes encoding known apoptotic regulators. Hence, it represents a sensitized background that can be used to screen for mutations that have a significant impact on dE2F1-induced apoptosis.

### Identification of Aac11 as a Modifier of dE2F1-Dependent Phenotypes

We screened a collection of recessive-lethal P-element transposon insertions for mutations that modified the *Act88F-Gal4,UAS-dE2f1* wing phenotype (see Materials and Methods). Dominant modifiers were retested against four other dE2F1-dependent phenotypes that were generated in the eye and in bristle cells and were retested against other apoptotic phenotypes to assess the specificity of the interactions (see below). The primary question arising from this type of screen was whether the modifiers isolated in this way do genuinely, and specifically, affect E2F-dependent apoptosis. Therefore, as described below, we have taken one such modifier and have characterized the interaction in detail.

**Figure 4:**
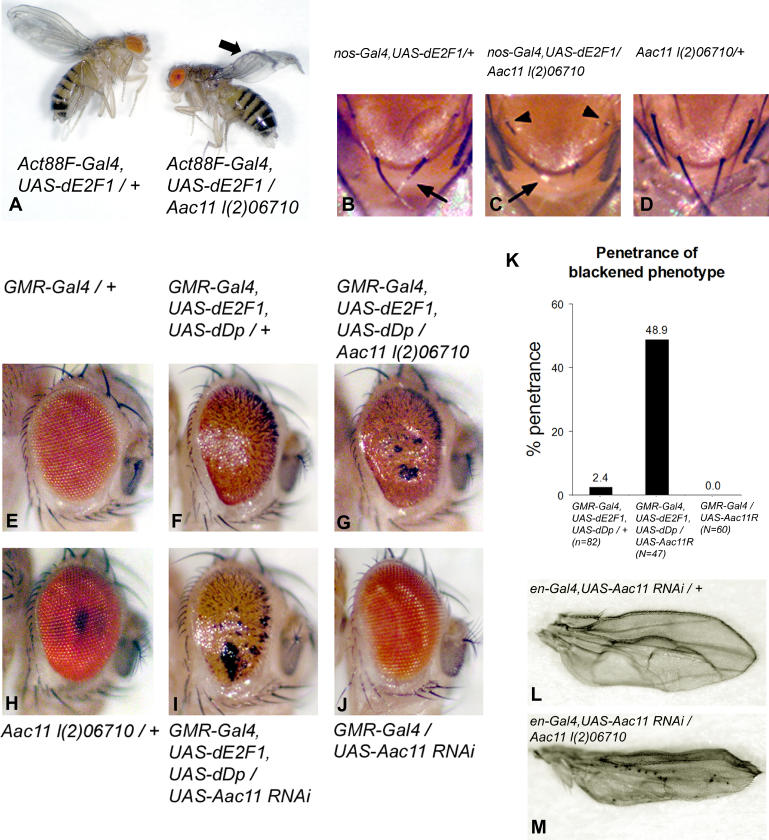
*Aac11* Loss-of-Function Specifically Enhances Multiple *dE2f1*-Dependent Phenotypes In Vivo Through genetic screening, the P-element insertion *l(2)k06710* was identified as a strong enhancer of the *Act88F-Gal4;UAS-dE2f1* apoptotic wing phenotype (A). The arrow in (A) indicates enhanced ventral wing curvature and blistering in the *l(2)k06710-*enhanced *dE2f1*-dependent wing phenotype. In secondary screening, *l(2)k06710* was found to enhance multiple *dE2f1*-dependent phenotypes in different tissues including a *nos-Gal4;UAS-dE2f1* notum bristle phenotype (B–D) and a *GMR-Gal4,UAS-dE2f1,UAS-dDp* rough eye phenotype (E–H). The *l(2)k06710* insertion enhanced bristle degeneration (compare arrows in [B] and [C]) and bristle stubble (arrowheads in [C]) induced by *dE2f1* without inducing bristle phenotypes under heterozygous conditions alone (D). Expression of a *UAS-Aac11 RNAi* allele also enhanced the *dE2f1*-induced rough eye resulting in a blackened phenotype (I–K). Expression of the *UAS-Aac11 RNAi* allele under engrailed results in dominant posterior wing blistering which is enhanced by the *l(2)k06710* P-insertion (L and M).

**Figure 5:**
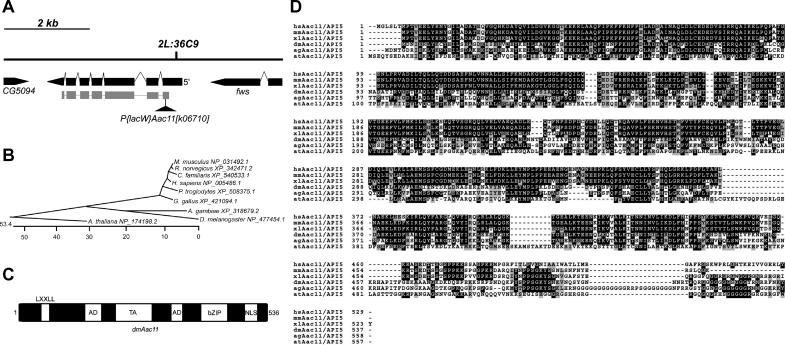
*Aac11* Is a Member of the Api5 Protein Family (A) P-element *l(2)k06710* insertion in the *Drosophila Aac11* gene. The genomic locus of *Aac11* on Chromosome 2L depicting P-insertion *l(2)k06710* (+187 nucleotides) in *Aac11* exon1. This transposon failed to complement *Df(2L)H20* covering region 36C9 but not the nearby deficiency *Df(2L)VA18*. (B) Phylogenetic tree of Api5 family proteins. Nine Homologene-annotated homolog sequences (NCBI) were aligned using MegAlign PhyloTree (DNAStar software, http://dnastar.com), using a Clustal method with PAM250 residue weight table. Additional species expressed-sequence tags are present but not included here. (C) Schematic of conserved Api5 protein domains. (D) ClustalW multiple alignment of human, mouse, frog, fly, mosquito, and plant Api5 homologs with gray and black depicting amino acid similarity and identity, respectively.

The P-element insertion *l(2)k06710* was a strong and specific enhancer of dE2F1-dependent phenotypes in the wing, eye, and bristles (Figure 4). The recessive-lethal *l(2)k06710* insertion failed to complement the genomic deficiency *Df(2L)H20,* and a similar set of interactions were observed using this deletion (unpublished data). *Df(2L)H20* spans 36A8–9 to 36F1 of Chromosome 2L and uncovers the *l(2)k06710* insertion site within the first exon of the *Drosophila Aac11* gene (Figure 5).

*Aac11* is a member of the *API5* gene family (Figure 5B). Alignment of the human, mouse, frog, fly, mosquito, and plant *API5* homolog products shows a high level of conservation throughout the majority of the protein, as well as a number of conserved protein domains (Figure 5C and 5D and Discussion). Interestingly, the human *Aac11/API5* gene, also known as *AAC-11/API5L1/FIF/MIG8* and hereafter referred to as *API5* (NCBI Homologene), is located at Chromosome 11p12–13 in a region frequently associated with chromosomal aberrations including amplification in glioma and breast tumor cells [34,35]. Api5 is an antiapoptotic protein first described in a mammalian cDNA screen for prosurvival genes; its expression promoted long-term cell survival in the absence of serum [36]. Api5 expression has been linked to tumorigenesis in a number of studies, although its function is unknown [36–39].

The interaction between *dE2f1* and *Aac11* is highly specific; *l(2)k06710* did not modify rough eye phenotypes generated by the expression of *rpr, hid, dp53, CycE,* or *p21* (Table 1). In addition, the effects of the *Aac11* insertion on dE2F1 are most likely downstream of RBF1 since the *l(2)06710* insertion did not modify phenotypes from *Rbf* transgenes (Table 1). *l(2)k06710* had no effect on an *Act88F-Gal4,UAS-ced3* wing phenotype that phenocopies *Act88F-Gal4,UAS-dE2f1* (Table 1)*,* demonstrating that the mutation does not indirectly affect the *Act88F* promoter or some aspect of Gal4 function. As control, the *Act88F-Gal4,UAS-ced3* phenotype was totally suppressed by coexpression of *p35* but unaffected by *Rbf* or dominant-negative *dDp* (Figure S1).

**Table 1:**
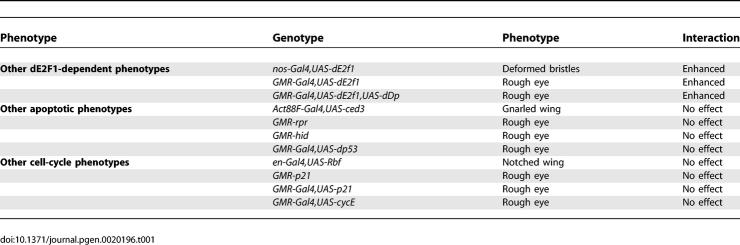
Genetic Interaction of *l(2)k06710* in Secondary Screen Phenotypes

Tissue-specific RNAi was used to confirm that these interactions were attributed to reduced levels of Aac11. An inverted repeat specific to *Aac11* (exons 1–3) was cloned downstream of UAS binding sites [40], and transgenic *UAS-Aac11 RNAi (UAS-Aac11R)* alleles were established. When *UAS-Aac11R* alleles were combined with various wing Gal4 drivers *(engrailed, apterous,* and *Act88F),* we observed dominant gnarled and blistered wing phenotypes that resembled the effects of dE2F1 expression (Figure 4L). These effects were dramatically enhanced by the presence of a single *l(2)k06710* allele (Figure 4M). When tested in the eye, expression of *UAS-Aac11R* gave no phenotype alone but strongly enhanced the blackened and rough eye phenotypes caused by *GMR*-regulated expression of *dE2f1* and *dDp* (Figure 4I–4K). Blacked areas of the eye have been previously described (*burned* and *scorched* phenotypes) and are characterized by pupal disc neurodegeneration [41]. Taken together, these results indicate that RNAi-mediated depletion of Aac11 is sufficient to enhance dE2F1-induced phenotypes as well as to induce dominant phenotypes in the fly wing.

### Aac11 Inhibits dE2F1-Dependent Apoptosis without Generally Affecting dE2F1 Transcriptional Activity

**Figure 6:**
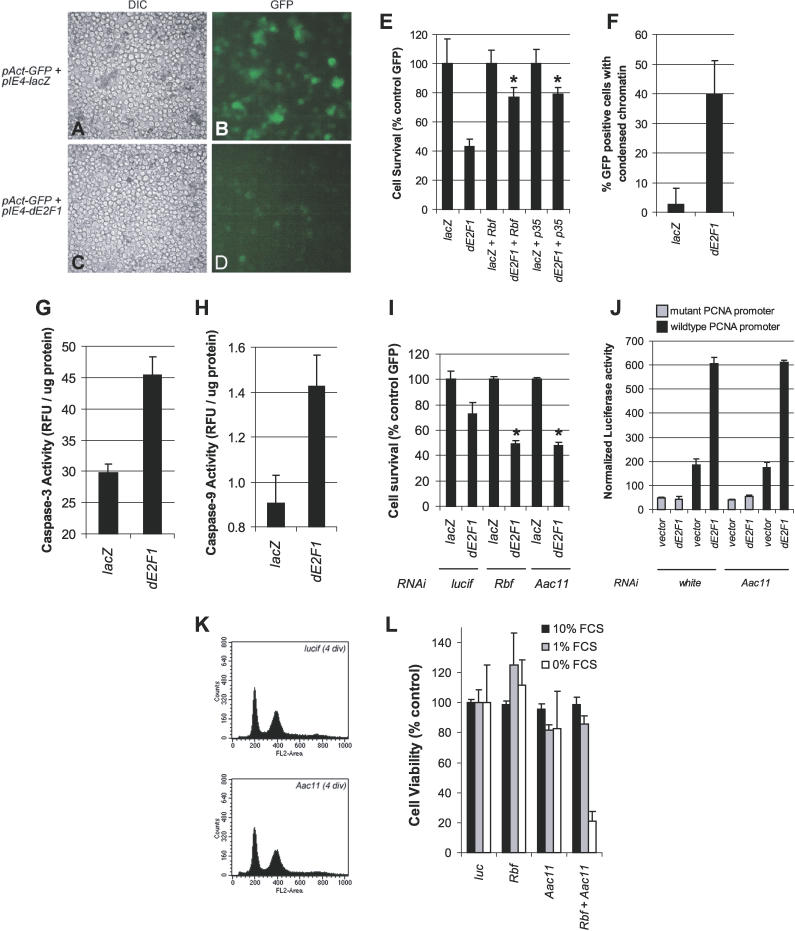
RNAi of Aac11 Enhances dE2F1-Induced Apoptosis and Is Synthetic-Lethal with RBF1 RNAi (A–D) Transfection of dE2F1 in *Drosophila* SL2 cells induces cell death as determined by co-transfected GFP loss. (E) Quantitative measurements of GFP in dE2F1 transfections demonstrated significant GFP loss from dE2F1 that could be rescued by either RBF1 or p35 cotransfection (*p < 0.05 by *t*-test). Transfection of dE2F1 induced (F) apoptotic chromatin condensation in DAPI-stained nuclei in GFP-positive cells, (G) caspase-3 activation, and (H) caspase-9 activation 48 h after transfection. (I) RBF1 or Aac11 RNAi significantly enhanced dE2F1-dependent apoptosis (*p* < 0.01 by *t*-test). Cell survival was determined by GFP assay 48 h after transfection. (J) Aac11 RNAi does not affect dE2F1 transcriptional activation of the *Drosophila PCNA* promoter. (K) Aac11 depletion does not alter cell cycle profiles in SL2 cells as determined by flow-cytometry analysis (div = days in vitro after RNAi). (L) Aac11 RNAi is synthetic lethal with RBF1 RNAi under conditions of low-serum stress. Cells were treated with dsRNA in serum-free media and split into media with and without serum, and cell survival was determined 3 d after RNAi.

In addition to apoptosis, wing gnarling and blistering can be induced by a variety of different mechanisms that include changes in cell fate, adhesion, and proliferation. To confirm that Aac11 affects dE2F1-dependent apoptosis, rather than simply causing a synergistic disruption in tissue development, we moved away from the context in which we had discovered the connection between Aac11 and dE2F1 and reconstructed this genetic interaction in cultured *Drosophila* cells (Figure 6). A dE2F1-expression construct, or lacZ as control protein, was introduced into SL2 cells together with a GFP-expression construct that allowed us to visualize the transfected cells. As expected from the proapoptotic activity of dE2F1, very few GFP-positive cells were found in dE2F1-transfected cultures compared to the lacZ control after 48 h (Figure 6A–6D). The level of GFP expression was measured by fluorimetry, and this enabled the extent of dE2F1-induced cell killing to be quantified (Figure 6E). The effects of dE2F1 were both time and dosage dependent and quantitatively similar to the changes seen when the proapoptotic *Drosophila* gene, *hid,* was expressed as a positive control (unpublished data). As expected, the effects of dE2F1 in this assay were inhibited by the coexpression of RBF1 (Figure 6E).

Three lines of evidence confirmed that the loss of GFP in this assay was due to E2F1-induced apoptosis. First, a significant increase in the number of Hoechst 33258–positive apoptotic chromatin-condensed nuclei was observed following dE2F1 expression, compared to lacZ-transfected controls (Figure 6F). Second, transfection of dE2F1 induced the activation of both initiator and effector caspases (Figure 6G and 6H). Third, the effects of dE2F1 were blocked by the coexpression of either RBF1 or the baculovirus caspase inhibitor p35 (Figure 6E).

Using this assay, we asked whether Aac11 activity was a limiting factor for dE2F1-induced apoptosis by measuring the level of dE2F1-induced death in cells depleted of Aac11 by RNA interference (RNAi). Cells were treated with luciferase double-stranded RNA (dsRNA) as nonspecific control, RBF1 dsRNA as positive control, or Aac11 dsRNA for 3 d and then cotransfected with GFP and either lacZ or dE2F1 (along with each dsRNA, respectively). As expected, depletion of RBF1 significantly enhanced dE2F1-dependent death compared to lacZ-transfected controls (Figure 6I). Depletion of Aac11 also enhanced dE2F1-induced apoptosis to a level that was comparable to that caused by the depletion of RBF1 (Figure 6I). Aac11 depletion alone did not result in growth or cell cycle phenotypes under these conditions (Figure 6K), indicating that the changes were unlikely to result from a nonspecific effect on cell number. These data indicate that endogenous Aac11, like RBF1, suppresses the apoptotic activity of dE2F1.

To ask whether Aac11 might repress the transcriptional activity of dE2F1, we tested whether depletion of Aac11 by RNAi altered dE2F1′s ability to activate transcription. *Drosophila* SL2 cells were treated with Aac11 dsRNA, or nonspecific dsRNA to the *white* gene as control for 5 d, and then transiently transfected with a wild-type or mutant *PCNA-lucif* reporter construct. The reporter was titrated to submaximal levels to ensure that either an increase or a decrease in transcription could be measured. As expected, dE2F1 transfection activated transcription from the wild-type *PCNA* promoter but not a promoter with mutant E2F binding sites (Figure 6J). The wild-type *PCNA* promoter, but not the E2F-binding mutant, is activated by RBF1 RNAi [27]. However, no change in dE2F1-mediated activation was observed in Aac11-depleted cells (Figure 6J). This indicates that Aac11 depletion alters dE2F1-dependent apoptosis without generally elevating dE2F1-dependent transcription; hence, Aac11 most likely acts downstream of dE2F1-mediated transcriptional activation. Although these data suggest that Aac11 does not generally affect E2F-dependent transcriptional activity, we cannot rule out potential effects of Aac11 on a subset of E2F promoters.

To test the idea that Aac11 affects dE2F1 in a manner that is distinct from RBF1, we examined the effects of depleting both Aac11 and RBF1. No significant changes in cell survival or proliferation were observed following the depletion of either RBF1 or Aac11, or both, in the presence of serum (Figure 6L). However, SL2 cells are more sensitive to apoptosis following serum deprivation. In these conditions, cells that would normally survive if Aac11 and RBF1 were depleted individually died when both proteins were depleted (Figure 6L). This synthetic lethality confirms that Aac11 and RBF1 have independent functions and raises the possibility that Aac11 may act generally to protect RBF1-deficient cells from E2F-induced apoptosis in contexts where survival signals are limiting.

### Api5 Suppresses E2F1-Induced Apoptosis in Human Tumor Cells, and Api5 Depletion Is Tumor Cell Lethal

**Figure 7:**
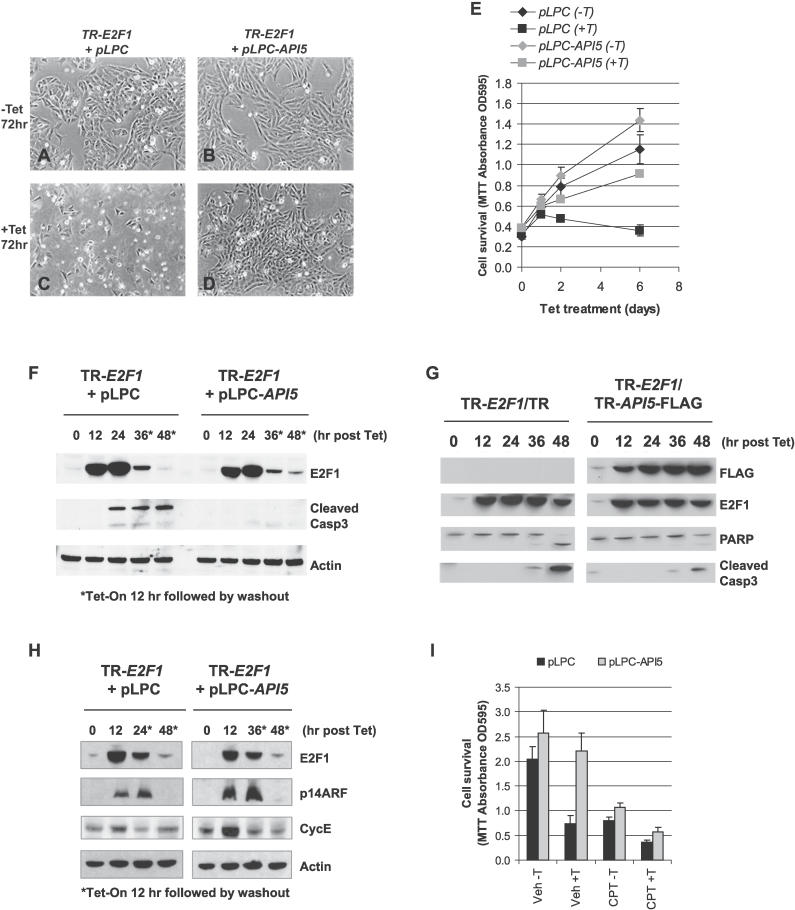
Human Api5 Specifically Abrogates E2F1-Dependent Apoptosis without Generally Affecting E2F1-Dependent Transcription (A–D) *API5* stably expressing Saos-2 cells were generated with a Tet-inducible *E2F1* transgene in the background. Following E2F1 induction by Tet treatment, the parental cells undergo rapid widespread apoptosis; however, the Api5-expressing cells are highly resistant to E2F1-induced cell death. (E) Api5-expressing cells survive and proliferate even following high and sustained levels of E2F1 expression. Cells were grown for 6 d after Tet re-dosing every other day. (F and G) Api5 reduces the levels of E2F1-mediated caspase-3 and PARP cleavage in both stable and Tet-inducible Api5 Saos-2 cells. (H) Api5 expression does not inhibit the E2F1-mediated induction of target genes *CycE* and *p14^ARF^* in Saos-2 cells. (I) Api5 expression blocks death induced by E2F1 (+T) but not by treatment with the DNA-damaging agent camptothecin (CPT) as compared to DMSO vehicle control (Veh). Saos-2 cell survival was assayed at 48 h by MTT.

An underlying premise to this work is the idea that our understanding of E2F-dependent apoptosis is incomplete. Since much of the current information about E2F is derived from studies in mammalian cells, it is important to know whether novel functional interactions discovered in a genetic screen in *Drosophila* are also relevant to studies of the mammalian factor. To test the evolutionary conservation of the genetic interaction between dE2F1 and Aac11, we examined the effects of their homologs in human cells. Lines of Saos-2 cells, a human osteosarcoma cell line that is both *Rb* and *p53* deficient, were generated containing a tetracycline (Tet)-responsive transgene controlling human *E2F1* expression. A cDNA for the human *API5* gene was cloned and used to generate paired cell lines, with or without exogenous Api5. Using these lines, we examined the effects of elevated E2F1 (Figure 7).

E2F1 expression in Saos-2 cells causes extensive apoptosis (Figure 7A–7D) beginning at approximately 30 h postinduction and preceded and accompanied by elevated expression of various E2F target genes such as *p14^ARF^* and *CycE* (Figure 7H). Tet-induction in this system results in significant elevation of E2F1 over endogenous as observed by Western analysis (Figure 7F–7H). Remarkably, the overexpression of Api5 strongly inhibited E2F1-induced apoptosis (Figure 7A–7D), giving significantly enhanced cell survival even when very high levels of E2F1 expression were sustained for over 1 wk in culture (Figure 7E). Accordingly, the ability of E2F1 to induce levels of apoptotic caspase-3 and PARP cleavage was abrogated by exogenous Api5 (Figure 7F and 7G). However, the expression of Api5 did not prevent E2F1-mediated induction of the E2F target genes *p14^ARF^* and *CycE* (Figure 7H). Thus, in human cells, as in *Drosophila,* Api5 suppresses E2F-induced apoptosis and most likely acts downstream of E2F-induced transcription, although we cannot rule out effects on other promoters at the moment.

To explore the specificity of Api5 action, control or Api5-expressing Saos-2 cells were treated with or without tetracycline, to induce E2F1, in the presence or absence of the DNA-damaging agent camptothecin. Whereas Api5 protected cells from E2F1-induced death, it failed to protect against apoptosis induced by camptothecin (Figure 7I). Moreover, the ability of Api5 to protect against E2F1-induced death was overridden by camptothecin treatment. Expression of Api5 was also insufficient to block death induced by vinblastine, staurosporine, rotenone, or tumor necrosis factor-alpha and had no effect on cell death induced by the retroviral expression of p53 or p73 (unpublished data). Hence, in human cells, as in *Drosophila,* Api5 proteins provide a very specific protection against E2F-induced apoptosis, and its protective activity distinguishes between these paradigms of cell death.

**Figure 8:**
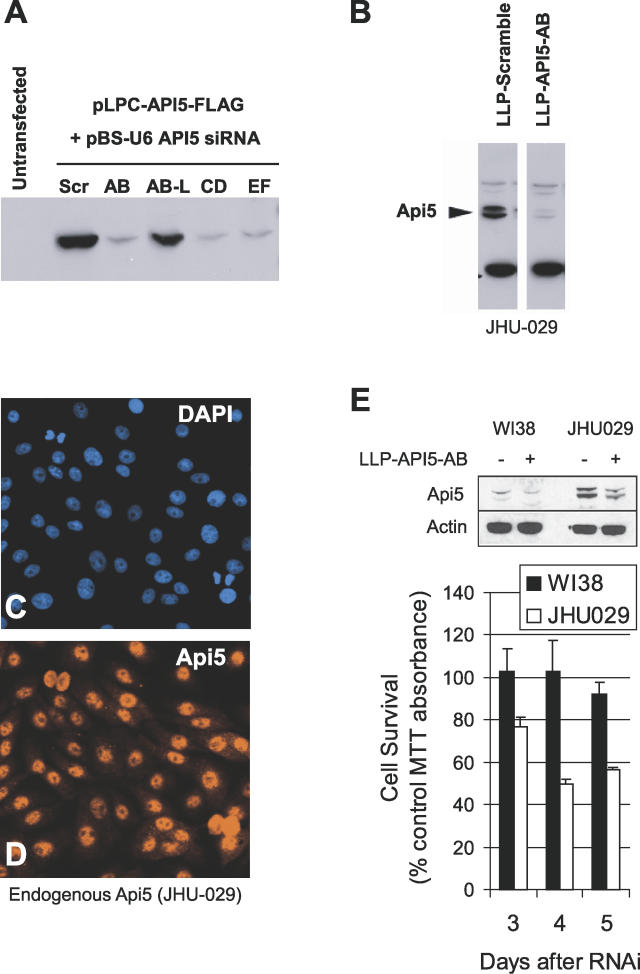
Depletion of Human Api5 in p16^INK4a^-Deficient Squamous Cell Carcinoma Cells Results in Reduced Survival versus Normal Human Fibroblasts (A) RNAi-mediated depletion of transfected Api5. Various shRNA constructs were tested for their ability to deplete FLAG-Api5 transfected U2OS cells. The AB, CD, and EF (but not Scramble or AB-L) cotransfected constructs strongly depleted Api5 expression after 3 d as determined by anti-FLAG immunoblot. (B) RNAi-mediated depletion of endogenous Api5 protein. JHU-029 cells were infected with lentiviral encoding scramble or API5-AB shRNAs and selected with puromycin for 3 d. Expression of endogenous Api5 was determined by immunoblotting with affinity-purified anti-Api5 polyclonal antibody (G3162). (C and D) Endogenous Api5 expression in JHU-029 cells is nuclear and excluded from the nucleolus. JHU-029 cells were stained with the G3162 polyclonal antibody after 4% paraformaldehyde fixation. (E) Api5 RNAi reduces survival of JHU-029 tumor cells as compared to normal human fibroblasts. JHU-029 cells, as well as normal human WI38 diploid fibroblasts, were infected with lentiviral empty vector or vectors encoding API5-AB shRNAs for 24 h and plated onto culture plates in 10% fetal calf serum–containing media. Cell survival was determined by MTT assay at the indicated days post–lentiviral infection. Api5 and control actin expression was determined from equally loaded protein from day 3 lysates.

Tumor studies have shown that Api5 is preferentially expressed in squamous cell carcinoma versus adenocarcinoma in non–small cell lung cancer [38]. We hypothesized that endogenous Api5 might be an important regulator of survival in squamous cell carcinoma and tested this by reducing Api5 expression (Figure 8). shRNA constructs to Api5 were designed, tested for their ability to deplete transfected FLAG-tagged Api5 (Figure 8A), and then expressed from lentiviral vectors (LLP) to target the endogenous Api5 protein (51-kDa doublet) in human squamous cell carcinoma 029 cells (JHU-029) (Figure 8B). JHU-029 cells are deficient for p16^INK4a^ [42] and endogenously express nuclear-localized Api5 (Figure 8C and 8D). Compared to control WI38 human diploid fibroblasts, endogenous Api5 is highly expressed and RNAi depletion of Api5 resulted in reduced survival with higher sensitivity in the tumor cells (Figure 8E). In keeping with the synthetic lethality between RBF1 and Aac11 depletion in SL2 cells, apoptosis was even more evident when Api5-depleted cells were maintained in low-serum (unpublished data). Taken together, the extensive pattern of genetic interactions between E2F1 and Api5, and the conservation of these interactions from flies to humans, underscores the significance of Api5 for E2F1-dependent apoptosis. Depletion of Api5 in E2F-deregulated tumor cells results in reduced survival, and this raises the possibility that Api5 may be a useful target for antineoplastic therapy.

## Discussion

E2F-dependent apoptosis has been implicated in a wide variety of pathophysiological settings, including DNA damage signaling, neurodegeneration, and in the consequences of pRB inactivation in cancer cells (for review, see [5,43,44]). Very little is known about the regulation of E2F-dependent apoptosis in vivo; most of our current knowledge comes from studies of cultured cell lines. Because of this paucity of information, we predict that many of the genes that have the greatest impact on E2F-dependent apoptosis in vivo have yet to be identified.

Here we describe a series of tools for the study of E2F-induced apoptosis in *Drosophila*. Placing dE2F1 expression under the control of the *Act88F-Gal4* driver induces premature apoptosis in the developing wing, giving a gnarled and blistered wing phenotype. These effects are dosage sensitive and can be modified not only by changing the levels or activity of dE2F1 but also by coexpressing regulators of cell death and by heterozygous mutations in genes known to function in cell death pathways. Indeed, similar phenotypes can be generated by the misexpression of proapoptotic genes from the same driver.

We note that *Drosophila* may be particularly advantageous for the study of E2F-induced apoptosis. Since flies have only one activator *E2F* gene, and one *DP* gene, the way that signaling pathways converge on E2F may be easier to dissect in *Drosophila* than in mammalian cells (eight *E2F* and three *DP* genes have been described to date). Several of the known features of E2F1-induced apoptosis in mammalian cells are conserved in flies. For example, in the *Drosophila* wing, we find that dE2F1-dependent death is regulated by both dArk/Apaf1-dependent apoptosome function and requires downstream effector caspase activity as reported in mammalian settings in vivo [20,45,46]. Moreover, we also found that this activity is dominantly modified by dIAP1 in vivo*.* However, some aspects of the mammalian interactions do not appear to be present. For example, we failed to find any evidence for genetic interactions between *dE2f1* and *dp53*. Several different pathways for E2F-induced apoptosis have been proposed for mammalian cells, and the *Drosophila* model may most closely resemble p53-independent forms of apoptosis induced by the mammalian E2Fs. Understanding mechanisms of p53-independent apoptosis by E2F1 is particularly important given the high incidence of *p53* mutations in human tumors.

While the *Act88F-Gal4,UAS-dE2f1* wing phenotype provides a sensitized background to identify modifiers of E2F-dependent apoptosis, modification of this phenotype does not necessarily implicate a gene in dE2F-induced death. Mutations that have synergistic or antagonistic effects on the processes of wing development that are disrupted by dE2F1 will also change the severity of the phenotype. In this case, the genetic interaction would inform us about the context in which dE2F1 is being expressed but would not give us insight into specific activities of E2F. A related, but different, issue is the possibility that a mutation that affects cellular sensitivity to apoptosis acts in general, rather than being specific to the process of E2F-induced apoptosis. We therefore designed a series of secondary assays to distinguish between classes of modifiers.

One way to eliminate developmental context as the reason for a genetic interaction is to look for interactions in different tissues and different stages of development. We found that *UAS-dE2f1* expression from three additional drivers *(sca-Gal4, nos-Gal4, GMR-Gal4)* gave visible phenotypes that could also be suppressed by coexpression of RBF1, dominant-negative dDP, or baculovirus p35 (*GMR-Gal4, nos-Gal4*; the *sca-Gal4* phenotype is too severe). These phenotypes can also be dominantly modified by heterozygous mutations, and we infer that mutants that genetically interact with dE2F1 in multiple different contexts are more likely to be informative. Of course, a potential weakness of this rationale is that if E2F-dependent apoptosis is controlled by tissue specific mechanisms, then the genetic interactions might only occur in one specific context. As an alternative strategy that completely removes any potential contribution from developmental context, we have also established assays for dE2F1-induced apoptosis in tissue-culture cells. This assay system allows candidate genes to be tested by both overexpression and loss-of-function approaches (RNAi). In addition, the tissue culture assays allow effects on the level and rate of dE2F1-dependent apoptosis to be quantified precisely and open the way to more mechanistic studies of genetic interactors.

Mutations that specifically affect dE2F1-induced apoptosis could be distinguished from mutations that modify apoptosis in general using *Act88F-Gal4,UAS-ced3*. Heterologous misexpression of this C. elegans caspase from the same *Act88F-Gal4* driver gave a phenotype that was very similar to *Act88F-Gal4,UAS-dE2f1* but was insensitive to changes in E2F activity. As an alternative, *Act88F-Gal4,UAS-human caspase-1* could also be used for this purpose (unpublished data). Other transgenes such as *GMR-hid* and *GMR-rpr* provided additional tests for specificity, using specific proapoptotic molecules and the eye rather than the wing as the context to score interactions.

Using these tools, we made the unexpected finding that Api5 proteins are important and specific determinants of E2F-induced death. As described here, the evidence for a functional connection between E2F and Api5 is compelling: (1) mutation of *Aac11* enhances the phenotypes caused by elevated levels of dE2F1 in the *Drosophila* wing, eye, and bristles, (2) RNAi-mediated inhibition of Aac11 enhances dE2F1-induced phenotypes in vivo and in tissue culture cells, (3) RNAi depletion of Aac11 not only enhances dE2F1-induced apoptosis but also is synthetic-lethal with depletion of RBF1, (4) raising the level of Api5 strongly suppresses E2F1-induced apoptosis, and (5) depletion of Api5 is specifically lethal to tumor cells with deregulated E2F. These genetic interactions are relatively specific in both *Drosophila* and human cells; manipulating the levels of Api5/Aac11 did not affect apoptosis induced by caspases, *hid, rpr, p53,* or DNA-damaging agents.

*API5* was initially isolated as a gene whose expression promoted cell survival following serum deprivation [36]. Multiple studies have shown that the *API5* mRNA transcript is strongly expressed in transformed cell lines [36,37,47,48]. Consistent with this, we also found Api5 protein levels significantly elevated in tumor cell lines with known lesions in the pRB pathway (JHU-029 cells versus WI38 fibroblasts in Figure 7E and unpublished data). Recently, Api5 expression was reported to be repressed by *myb* [49] and activated by mutant *p5*3 [39]. In this latter study, *API5* was one of a cluster of genes that were upregulated by three different dominant gain-of-function tumor-derived *p53* missense mutants. Intriguingly, in these cells, whereas wild-type *p53* repressed Api5, the *p53* mutant alleles significantly activated Api5 expression. Therefore, the *API5* promoter may be specifically deregulated in tumors cells harboring dominant *p53* mutations. In addition to its survival-promoting activity, Api5 overexpression has been reported to induce cervical tumor cell invasiveness, and its expression has been found to be upregulated in some metastatic lymph node tissues [37], raising the possibility that it may be a metastatic oncogene. Api5 expression has been linked to poor prognosis in non–small cell lung cancer, and particularly in squamous cell carcinoma [38].

The discovery that Api5 is a potent suppressor of E2F1-induced apoptosis adds new significance to these observations. The genetic interactions described here suggest that Api5 may contribute to human malignancy by limiting the extent of E2F1-dependent cell death, and we suggest that this activity is particularly important when cells need to survive under suboptimal conditions. The synthetic-lethality observed with Aac11 and RBF1 depletion in low serum further suggests that Api5 might also promote the survival of tumor cells harboring pRb-inactivating mutations. Identification of new synthetic-lethal interactions is an important goal in developing cancer-specific therapies that should, in theory, reduce toxicity to normal cells [50]. Accordingly, RNAi-mediated depletion of Api5 resulted in enhanced cell death of *p16*-deficient squamous cell carcinoma cells, as compared to normal human fibroblast controls. Although future studies will be necessary to fully characterize the crosstalk between E2F and Api5 signaling pathways, these findings indicate that the levels of Api5 are likely to be very important for the survival of human tumor cells with deregulated E2F. Hence, Api5 may be an exploitable target for antineoplastic treatment, particularly in tumors with pRb inactivation.

Future experiments are now necessary to identify the molecular functions of Api5/Aac11. Orthologs of the *API5* gene family are highly conserved in species as diverse as plants and humans, but there are no obvious family members in worms or yeast. Api5 proteins share a number of conserved domains including a putative transactivation-domain flanked by two acidic domains, an LxxLL motif, a putative leucine zipper domain, and a nuclear localization sequence. The presence of these motifs suggests that Api5 family proteins might be transcriptional regulators. Various deletion mutants of Api5 possess strong transactivation activity when fused to the DNA binding domain of Gal4 [47]. However, to date, no target genes for Api5 have been described.

Perhaps one of the greatest advantages of the *Drosophila* system is the opportunity for broad-based genetic screens, and the tools described here allow novel interactors to be quickly characterized and categorized. Further screening with *Act88F-Gal4,UAS-dE2f1* could reveal additional mutations that, like Api5/Aac11, have a major impact on E2F-induced apoptosis in vivo. One of the most interesting aspects of the E2F–Api5 genetic interaction is the finding that it has been conserved between flies and humans during evolution. The discovery of the connection between Api5 and E2F underscores the point that although molecular studies have provided a great deal of information about the E2F-transcriptional program, not all of the genes that have a major impact on E2F-induced apoptosis in vivo have been identified. These results illustrate the need for genetic screens for mutations that have a significant impact on E2F-induced apoptosis and highlight the potential that components isolated in this way may be highly relevant in other species.

## Materials and Methods

### Fly transgenes, stocks, and crosses.

Unless otherwise noted, all fly crosses were conducted at 25 °C and phenotypes are depicted from female progeny. The initial Gal4 screen was conducted by crossing approximately 50 unique Gal4 lines to four different *UAS-dE2f1* lines *(3^rd^, 2BX, 5AII,* and *3CII)* at 18 °C, 25 °C, and, in some cases, 30 °C. The recessive-lethal P-element transposon collection (approximately 2,200 lines) was a generous gift from Dr. Spyros Artavanis-Tsakonas [51] and was F1 screened through the *Act88F-Gal4,UAS-dE2f1* wing phenotype and rescreened against the battery of secondary screens described in the Results section. We isolated 30 strong suppressors and two strong enhancers from the primary screen and the secondary screens narrowed these to ten insertions, in four different loci, that interacted with at least two additional *dE2f1*-dependent phenotypes but failed to modify other apoptosis phenotypes. From these, mutations in three loci acted as suppressors, and mutation in one locus was an enhancer *(Aac11).* The following stocks were used for these studies (stock identification numbers): *w^1118^, CycA^03946^, CycE^AR95^, stg^01235^, Cdk1^2^, Cdk4^05428^, Cdk4^06503^, th^l(3)j5C8^, Aac11^k06710^* (10645), *Df(2L)H20* (3180), *Df(2L)VA18* (6105), *GUS-dp53^DN259H^, GUS-dp53^DNCt^, UAS-EGFP* (5431), *UAS-lacZ* (8529), *UAS-dE2f1,UAS-dDp* (4774), *nanos-Gal4* (4442), *GMR-Gal4* (1104), and *apterous-Gal4* (G2–1) (Bloomington stock center)*; UAS-ced3 (6–6), UAS-caspase-1 (7–1)* (Teiichi Tanimura); *UAS-dp53, dp53^4−^, dp53^3+^* (Michael Brodsky); *dArk^CD4^* (John Abrams); *engrailed-Gal4, sca-Gal4, UAS-p21, GMR-p21, UAS-dacapo* (Iswar Hariharan); *Act88F-Gal4 (2^nd^)* (Eric Fyrberg); *Cdk1^E10^, UAS-CycE* (Christian Lehner), *UAS-rpr, GMR-rpr, UAS-hid, UAS-grim, GMR-hid, UAS-p35, UAS-dIAP1* (Kristin White). The *UAS-Rbf (4)* stock was described previously [52]. The following double-balanced or recombinant stocks were created for these studies: (a) *Act88F-Gal4,UAS-dE2f1 (B2)/CyO ftz lacZ*; (b) *Act88F-Gal4,UAS-EGFP (A)/CyO ftz lacZ*; (c) *Act88F-Gal4,UAS-EGFP,UAS-dE2f1 (3A)/CyO ftz lacZ*; (d) *Act88F-Gal4,UAS-ced3 (4)/CyO ftz lacZ*; (e) *GMR-Gal4,UAS-dp53/CyO ftz lacZ*; (f) *GMR-Gal4/CyO*; *UAS-dE2f1/TM6b*; (g) *GMR-Gal4,UAS-dE2f1,UAS-dDp/CyO ftz lacZ;* (h) *Sca-Gal4/CyO, UAS-dE2f1/TM6b*; (i) *nos-Gal4,UAS-dE2f1/CyO ftz lacZ*; (j) *en-Gal4,UAS-Rbf/CyO, ftz, lacZ*; *(*k) *GMR-Gal4,UAS-p21/CyO ftz lacZ*; (l) *GMR-Gal4,UAS-CycE/CyO ftz lacZ*; (m) *en-Gal4,UAS-Aac11 RNAi (9A)/CyO ftz lacZ*; and (n) *en-Gal4,UAS-Aac11 RNAi (9A)/T(2:3) CyO TM6b*. The *UAS-dDp dominant-negative* allele (Chromosome 3) was created by subcloning the dDp^113–337^ amino-acid fragment into the *pUASt* vector [29]**.** The *UAS-Aac11 RNAi* allele was created by subcloning an inverted repeat of an XbaI fragment containing Aac11 5′ UTR-exon3 sequences (primer set, 5′- GCGCTCTAGAGCTGTCTCGAGATCTGGTCACTC and 5′-GCGCTCTAGAGCGTTTCCCTGGCACAGTTTC) into the *pWIZ* vector [40]. P-element transformation was performed as described [53]. All transgenic fly embryo injections were performed by the CBRC Transgenic Fly Core.

### AO staining and EGFP quantification in single flies.

AO staining of apoptotic cells in the wing was performed as described [54]. AO positive cells were visualized with fluorescent microscopy under FITC filters (excitation λ 490 nm, emission λ 520 nm) and pseudocolor-depicted. As positive control, we detected AO positive cells in the posterior compartment of newly eclosed wings from *en-Gal4/UAS-dp53* progeny (unpublished data). EGFP fluorescence in individual newly eclosed female flies was quantified in single flies as described [55]. EGFP fluorescence was determined at excitation λ 488 nm, emission λ 511 nm (cutoff λ 495 nm) with buffer background subtraction. Fluorescent measurements were determined within a linear range from a single *UAS-EGFP* transgene; two *UAS-EGFP* transgenes produced 2× relative fluorescent units (RFU) (unpublished data). We did not detect squelching since expression of control protein from a single *UAS-lacZ* transgene had no effect on EGFP fluorescence (unpublished data).

### Cell culture and transient transfections.

*Drosophila* Schneider line 2 (SL2 cells; ATCC, http://www.atcc.com) and mammalian cells were grown as previously described [52,56]. *Drosophila* cell (CellFectin reagent; Invitrogen, http://www.invitrogen.com) and human cell (Fugene-6 reagent; Roche, http://www.roche.com) transfections were performed according to the manufacturer's recommended instructions. Luciferase reporter assays were performed as described [52]. The wild-type and E2F-binding mutant *PCNA-luciferase* reporter was kindly provided [57].

### Apoptosis and viability assays.

Caspase-3 (DEVD-AFC peptide substrate) and caspase-9 (LEHD-AFC peptide substrate) enzymatic assays (R&D Systems, http://www.rndsystems.com) were performed according to the manufacturer's recommendations. Cleaved, activated fluorescent substrate was measured in RFU and normalized to total protein content (Bio-Rad, http://www.bio-rad.com). Cell viability was determined by 0.4% trypan blue exclusion hemocytometry or MTT assay [58]. Apoptotic chromatin condensation was assayed in GFP-cotransfected live cells by incubating cultures with membrane-permeant 50 μg/ml Hoechst 33258 dye (20 min; room temperature). Nuclei with apoptotic condensed chromatin was visualized under epifluorescence and scored in five nonoverlapping fields per condition expressed relative to total transfected. Transfected SL2 cell survival in six-well trays was determined by GFP cotransfection viability assay [59] with 0.3 μg/well pAct-GFP^US9^ (*Act5c* promoter) expression construct. Cells were harvested by trituration, pelleted by centrifugation, resuspended in PBS, and transferred to 96-well trays for GFP fluorescence quantification. Plasmids used for these studies were *pIE4-dE2f1, pIE4-Rbf, pIE4-lacZ,* and *pIE4-p35*.

### Flow cytometric analysis.

Cell cycle analysis using FACS CellQuest (Becton Dickinson, http://www.bd.com) of ethanol-fixed, propidium iodide–stained SL2 cells was performed as described [60].

### *Drosophila* SL2 cell RNAi.

All RNAi for *Drosophila* SL2 cells was performed as described [52]. Double-stranded RNA was synthesized with T7 RiboMax (Promega, http://www.promega.com). Cells were RNAi depleted using 50 μg of dsRNA for each gene and normalized with luciferase dsRNA for co-RNAi treatments. For RNAi-transfection experiments, 15 μg of dsRNA was included in each transfection after the initial RNAi depletion. Depletion of RBF1 was confirmed by Western analysis (unpublished data). Depletion of Aac11 RNA was confirmed on microarray analysis (unpublished data).

### Western analysis, antibodies, and immunocytochemistry.

Western blot and immunohistochemical analysis was performed using standard techniques. Antibodies used in this study include those against cleaved caspase-3 (9661; Cell Signaling, http://www.cellsignal.com), PARP (Ab2; EMD Biosciences, http://www.emdbiosciences.com),E2F1 (SC193; Santa Cruz Biotechnology, http://www.scbt.com), HA (Clone-11; Covance, http://www.covance.com),FLAG (M2; Sigma, http://www.signaaldrich.com), cyclin E (SC247; Santa Cruz Biotechnology), and p14^ARF^ (Ab2; NeoMarkers, Lab Vision Corporation, http://www.labvision.com). The Api5 polyclonal antibody was created by subcloning full-length human *API5* cDNA into pGEX, and GST-Api5 fusion protein was prepared and used to inject two rabbits for polyclonal production (Genemed Synthesis, http://www.genemedsyn.com). Two bleeds were screened against transfected tagged and untagged full-length Api5 to verify antigenicity. Positive bleeds were affinity-purified against PVDF membrane–bound GST-Api5, eluted with 100 mM glycine (pH 2.5), and Centricon-purified. Specificity of the pAB3162 affinity-purified antibody was confirmed using both transfected and endogenous Api5 with and without shRNA depletion of the specific bands.

### Creation of inducible E2F1 and stable Api5–expressing Saos-2 cells.

The Tet-inducible Saos-2 cell line was created by transfecting *pCDNA6-TR* (Invitrogen) into Saos-2 and selecting blasticidin (2.5 μg/ml)-resistant clones to create Saos-2-TR. The *E2F1* cDNA was cloned into *pCDNA4-TO* (Invitrogen) and transfected into Saos-2-TR, and blasticidin (2.5 μg/ml)- plus zeocin (100 μg/ml)-resistant clones were isolated and tested for induciblity with 0.1 μg/ml tetracycline. Saos2-TR-E2F1 was transformed with retrovirus containing either *pLPC* (a gift from Scott Lowe) or *pLPC* containing HA-tagged *API5* cDNA (cloned from human cDNA library) and selected with 1 μg/ml puromycin to create cells stably expressing Api5.

### shRNA construction and lentiviral infection.

A series of *API5* targeting shRNAs were created in *pBS-U6* as described [61]. Targeting sequences were as follows: *API5*-AB 5′-GGCCAGCATAAAGATGCCTAT-3′; *API5*-CD 5′-GGGTTGTTCAGCCAAATACTT-3′; *API5*-EF 5′-GGCCGACCTAGAACAGACCTT-3′. Sequences were subcloned from *pBS-U6* into Lentiviral vector *LLP,* and high-titer lentivirus was produced as previously described [62].

## Supporting Information

Figure S1 Genetic Characterization of a Recombinant *Act88F-Gal4,UAS-ced3* Transgenic Stock Various alleles were analyzed for modification of the *ced3-*dependent phenotype in trans*.* Coexpression of *p35* (C), but not *Rbf* (D) or *dominant-negative dDp* (E), completely suppressed the ced3 caspase wing phenotype. (F) The *l(2)06710 Aac11* mutant does not modify the ced3-dependent phenotype.
(1.2 MB PDF)

### Accession Numbers

The National Center for Biotechnology Information (NCBI) (http://www.ncbi.nlm.nih.gov) GeneID numbers for genes (and products) discussed herein are *H. sapiens API5* (8539), *CASP1* (834), *CASP3* (836), *E2F1* (1869), *TP53* (7157), *TP73* (7161), *CCNE1* (898), *CycE/CDKN1A* (1026), *ARF/CDKN2A* (1029), *RB1* (5925); *D. melanogaster E2f* (42550), *Dp* (36461), *Aac11* (35053), *Ark* (36914), *rpr* (40015), *cdk1/cdc2* (34411), *cdk2/cdc2c* (42453), *cdk4* (36854), *hid/W* (40009), *PCNA/mus209* (37290), *dIAP1/thread* (39753), *dap* (36001), *CycA* (39340), *CycE* (34924), *grim* (40014), *p53* (2768677), *Rbf* (31027), *stg* (43466), *Act88F* (41885); *C. elegans ced3* (178272); *baculovirus p35* (1403968).

## Abbreviations

Aac11: antiapoptosis clone-11
Api5: apoptosis inhibitor-5
AO: acridine orange; *CycE; cyclin E*
*dap*: *dacapo*
dIAP1: *Drosophila* inhibitor of apoptosis-1
*dp*: *dimerization partner*
dsRNA: Double-stranded RNA
E2F: E2-promoter binding factor
*EGFP*: *enhanced green fluorescent protein*
pRB: retinoblastoma protein
RNAi: RNA interference
*rpr*: *reaper*
Tet: tetracycline
