# Supplementary material for: Functional Identification of Api5 as a Suppressor of E2F-Dependent Apoptosis In Vivo
Source: PLoS Genet. 2006 Nov 17;2(11):e196. doi: 10.1371/journal.pgen.0020196 (PMC1636698; doi:10.1371/journal.pgen.0020196)

Morris et al., Top, Fig. S1

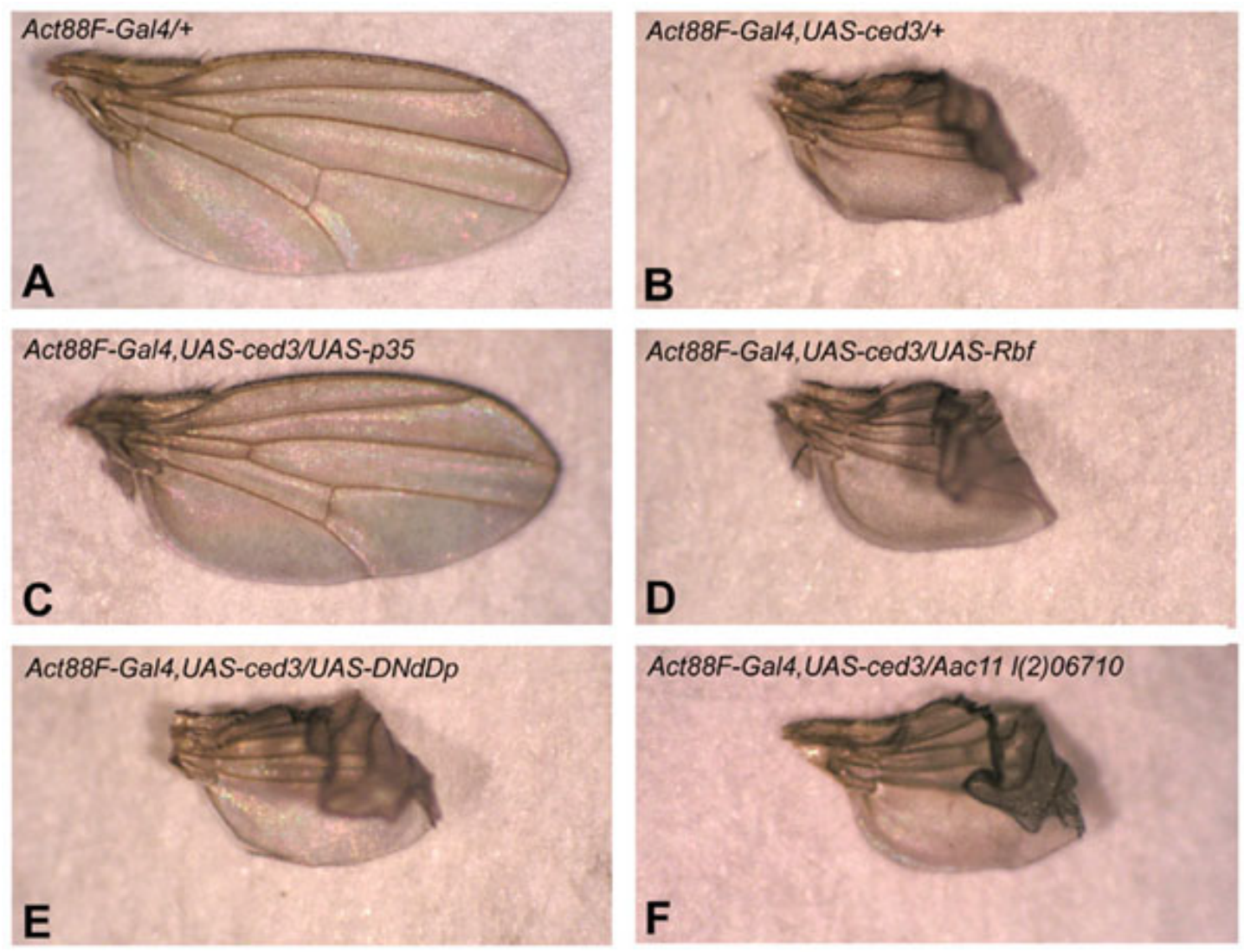

Supplement: Supplementary file 1 [file pgen.0020196.sg001.pdf]
